# Nutritional Management and Outcomes in Malnourished Medical Inpatients: Anorexia Nervosa

**DOI:** 10.3390/jcm8071042

**Published:** 2019-07-17

**Authors:** Cristina Cuerda, Maria F. Vasiloglou, Loredana Arhip

**Affiliations:** 1 Nutrition Unit, Instituto de Investigación Sanitaria Gregorio Marañón, Hospital General Universitario Gregorio Marañón, Calle del Dr. Esquerdo, 46, 28007 Madrid, Spain; 2 Diabetes Technology Research Group, ARTORG Center for Biomedical Engineering Research, University of Bern, Murtenstrasse 50, 3008 Bern, Switzerland

**Keywords:** anorexia nervosa, refeeding syndrome, weight gain, mortality, length of stay

## Abstract

Background: Anorexia Nervosa (AN) is a psychiatric disorder characterised by a physical and psychosocial deterioration due to an altered pattern on the intake and weight control. The severity of the disease is based on the degree of malnutrition. The objective of this article is to review the scientific evidence of the refeeding process of malnourished inpatients with AN; focusing on the clinical outcome. Methods: We conducted an extensive search in Medline and Cochrane; on April 22; 2019; using different search terms. After screening all abstracts; we identified 19 papers that corresponded to our inclusion criteria. Results: The article focuses on evidence on the characteristics of malnutrition and changes in body composition; energy and protein requirements; nutritional treatment; physical activity programmes; models of organisation of the nutritional treatment and nutritional support related outcomes in AN patients. Conclusion: Evidence-based standards for clinical practice with clear outcomes are needed to improve the management of these patients and standardise the healthcare process.

## 1. Introduction

AN is a psychiatric disorder characterised by a persistent restriction of energy intake leading to significantly low body weight, an intense fear of gaining weight or of becoming fat and a disturbance in the way one’s body weight or shape is experienced.

The diagnostic criteria of AN according to the latest edition of the Diagnostic and Statistical Manual of Mental Disorders (DSM-5) is represented in Table 1 [1].

The prevalence of AN is estimated to be 1% [2], with a ratio between women and men of 9:1. The age of onset of AN is usually adolescence or youth, although some cases appear after 40 years or in childhood.

This disease has a great tendency to chronicity, it can produce serious medical complications, it can interfere with the individual’s psychological and social development and it can even cause death. AN is associated with a high mortality rate (5%–10%) due to suicide, hydro-electrolyte alterations and arrhythmias, which places it at the head of psychiatric disorders [3].

Its aetiology is multifactorial and includes biological, psychological and cultural factors. Family and twin studies suggest a strong genetic component in AN. Within the biological factors are the neurochemical alterations [3,4]. The best known of these is the dysregulation of serotonin, which could also explain the high incidence of psychiatric comorbidities in these patients such as depression, anxiety and obsessive-compulsive disorders, which are associated with alterations in the serotonin system. Alterations have been found in these patients in some genes linked to different neurotransmitters (5HT2A receptor gene, MAOA monoamine oxidase gene, SERT serotonin transporter gene, NET noradrenaline transporter gene, among others) [4].

In addition, there are usually psychological problems and family dynamics. Patients with AN are described as anxious, depressive, perfectionists and with low self-esteem. Cultural influences are also important with the emphasis on thinness in our society, exacerbated by the media. All these factors can conclude in the appearance of AN in a vulnerable adolescent [5].

The treatment of AN requires a multidisciplinary team in which specialised professionals (physicians, nurses, dietitians, psychiatrists, psychologists, among others) work together. The treatment is based on the nutritional rehabilitation, management of complications, as well as on psychotherapy and psychotropic drugs [6].

The clinical manifestations of AN are multisystemic. A summary of them is presented in Table 2. The treatment of these alterations can be found in the literature [7,8].

The objective of this article is to review the scientific evidence of the refeeding process of malnourished inpatients with AN, focusing on the clinical outcome.

## 2. Methods

We conducted an extensive search in Medline and Cochrane, on April 22nd 2019, using the following search terms: “Anorexia nervosa”, “inpatients”, “refeeding”, “nutritional treatment”, “tube-feeding”, “energy expenditure”, “physical activity”, “exercise”, “refeeding syndrome”, “mortality”, “weight gain”, “rehospitalisation”, “length of stay”, in title and abstracts. We repeated the search using MeSH terms. We identified 259 papers. After screening all abstracts, we identified 19 papers that corresponded to our inclusion criteria. These were: (1) papers in English and Spanish in the last 15 years; (2) were performed in AN inpatients; (3) reported results on weight or body mass index (BMI) at admission and at discharge, length of stay, refeeding rate, type of nutritional treatment and outcome.

## 3. Results

As a result of the literature review, this article will cover the following topics: Characteristics of malnutrition and changes in body composition, energy and protein requirements, nutritional treatment, physical activity programmes, models of organisation of the nutritional treatment, and nutritional support related outcomes in AN patients.

### 3.1. Malnutrition and Body Composition in AN

According to the Global Leadership Initiative on Malnutrition (GLIM) classification, the patients with AN present chronic disease related malnutrition without inflammation with different degrees of severity. This means that the main etiological factor of malnutrition is starvation. However, if these patients suffer from an acute illness, the inflammatory process will rapidly deteriorate their nutritional status, jeopardising their clinical outcome [9].

Based on DSM-5 severity definition, patients with AN can be classified into mild BMI (BMI ≥ 17.0 kg/m^2^), moderate (BMI = 16.0–16.99 kg/m^2^), severe (BMI = 15.0–15.99 kg/m^2^) and extreme (BMI < 15.0 kg/m^2^) malnutrition [1].

The patients with AN may present with a low-fat mass and fat-free mass, however they usually maintain plasmatic visceral proteins in the normal range. It has been shown that low prealbumin is a predictor of medical complications [10].

The body composition of these patients can be evaluated with anthropometry, Bioelectrical Impedance Analysis (BIA) and Dual-Energy X-ray Absorptiometry (DEXA) as the gold standard. BIA has proved to be one of the most useful in clinical practice, although in severely malnourished patients (BMI < 15.0 kg/m^2^) may not be sufficiently accurate [11].

Phase angle is one of BIA measurements that has been associated with poor outcome in some diseases (HIV, cancer). In patients with low body weight, including healthy individuals, ballet dancers and AN patients, phase angle could be a useful marker of qualitative changes [12].

The use of BIA gives clinicians and researchers the advantage of monitoring the compartmental weight gain, ideally achieving an approximate 20/80%–25/75% fat/lean body mass ratio. Moreover, each patient can act as their own “control” that could potentially allow for a more effective, individualised nutrition regimen. Nevertheless, this information must be cautiously interpreted by qualified clinicians who understand this technique and its limitations [13].

The patients with AN present micronutrient deficiencies due to low food intake that can be aggravated in patients with purging habits and during the refeeding process. In a systematic review by Hanachi et al. including 374 patients with AN (restricting (AN-R) and binge-eating/purging (AN-BP) type), it was found that zinc deficiency had the highest prevalence (64.3%), followed by vitamin D (54.2%), copper (37.1%), selenium (20.5%), vitamin B1 (15%), vitamin B12 (4.7%), and vitamin B9 (8.9%). The AN-BP subgroup had lower selenium (*p* < 0.001) and vitamin B12 plasma concentration (*p* < 0.036), whereas lower copper plasma concentration was observed in patients with AN-R type (*p* < 0.022) [14].

### 3.2. Energy and Protein Requirements

The refeeding process of patients with AN can be challenging since it must allow a weight gain without developing a refeeding syndrome (RS). On top of that, physicians must deal with the difficult personalities of a psychiatric disorder which in this case shows itself as a fear of weight gain.

In order to obtain the energy requirements, there are equations that estimate the resting energy expenditure (REE) or basal energy expenditure (BEE) and methods that directly measure REE with different types of indirect calorimetry. All of these are applicable to clinical practice as well as research. Additionally, the use of indirect calorimetry can be used to monitor the nutritional treatment in hospitalised patients. Sometimes this helps to uncover patients that are not gaining weight accordingly.

In the study conducted by Cuerda et al., 22 female inpatients were studied. REE was measured by indirect calorimetry (Deltatrac II MBM-200, Aldershot, UK) and was estimated by several predictive formulas (Fleisch, Harris–Benedict, FAO, Schofield–HW, Schebendach). All formulas overestimated REE compared to indirect calorimetry, except the Schebendach formula [15].

In another study by El Ghoch et al., the REE measured by indirect calorimetry by the Douglas bag method and FitMate method was compared to the Harris–Benedict and Müller et al. equations in 15 patients with AN. Using the Douglas Bag method as the gold standard (that measures VCO2/VO2), the authors found an accurate REE estimation with the FitMate method and the Müller et al. equation. Meanwhile, the Harris–Benedict equation overestimated the REE [16].

The literature regarding protein requirements is scarce. There is no evidence that these patients have specific requirements for protein intake. The refeeding process is based on the recommendations for the general population (0.8 g/kg of body weight/day; 10%–15% of energy requirements).

### 3.3. Nutritional Treatment in AN

Nutritional rehabilitation is probably the most important part of the treatment of patients with AN. Its objectives focus on [6]: Restore body weight; correct the physical complications of malnutrition; improve the patients’ motivation to normalise their dietary habits and collaborate in the treatment; educate the patients about healthy nutrition and proper eating patterns; and correct wrong thoughts, attitudes, and feelings about the disorder.

Many of the cognitive and behavioural alterations of malnourished AN patients (food anxiety, taste alterations, binge eating, depression, obsessions, apathy, irritability) improve with the weight gain. Moreover, it increases the effectiveness of other treatments such as psychotherapy or psychotropic drugs [17].

Depending on the setting (outpatients, day hospital, inpatients), nutritional rehabilitation varies in terms of refeeding rate and type of nutritional treatment.

The restoration of body weight is done until the patient reaches a healthy weight in which women recover menstruation and ovulation, men normalise their sexual desire and hormone levels and children and adolescents normalise their growth and sexual development. This requires long treatments, sometimes with repeated hospitalisations followed by a continuity of care in the different settings.

The refeeding process is made according to the patient in a phased manner, agreeing on weight gains during hospitalisation and weight upon discharge. There are no clear criteria for hospitalisation. Some of the usual criteria are summarised in Table 3, even though this depends on the availability of a day hospital in the centre.

Generally, a weight gain of 0.5–1.4 kg/week in hospitalised patients and 250–500 g/week in outpatients is established to avoid the appearance of RS [6,17,18].

Table 4 summarises the recommendations of different clinical guidelines on how to perform the refeeding in malnourished AN patients. Since very few high evidence level studies (RCT) are available, these recommendations are based mainly on clinical experience [6,19,20,21,22,23]. These guidelines differ in the number of calories administered at the beginning of the refeeding (from the most conservative ones that recommend 5–20 kcal/kg at hospital admission [24], to the most permissive ones that begin with 30–40 kcal/kg) [6]. The caloric intake will increase progressively to allow adequate weight gain, sometimes being necessary to reach 70–100 kcal/kg. Patients who have higher caloric requirements usually present inappropriate behaviours (throwing or hiding food, vomiting, intense exercise, etc.).

An example of the progressive refeeding of macronutrients and micronutrients in hospitalised patients is shown in Table 5 [19].

In the last decades, many studies have been published regarding nutritional treatment in AN inpatients following different rates of refeeding. Some of them favour the side of “start slow, advance slow”, which translates to a slow rate of refeeding especially in patients with very low BMI at admission. On the other hand, some groups follow the “start higher, advance faster”, which means a more aggressive refeeding process generally in patients with moderate malnutrition with additional phosphate supplements. This usually conducts to a shorter hospital stay. In general, the results of these studies show that the refeeding is safe and effective in these patients if the supervision is adequate and follows a specific protocol [25,26,27,28,29,30,31,32,33,34,35,36,37,38,39,40].

**Figure 1:**
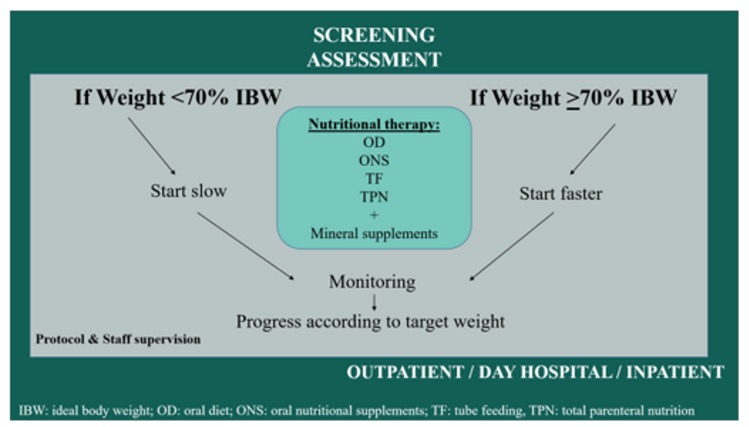
Algorithm of the nutritional treatment in AN.

By following any of the above recommendations the weight gain is progressive, and it avoids the appearance of RS (especially in patients weighing less than 70% of their ideal body weight). An algorithm of the nutritional treatment is shown in Figure 1.

The refeeding process can include oral diet (OD) as well as medical nutritional therapy.

OD should be the first option for the refeeding, even though there is no evidence of the best food choices and macronutrients distribution in the diet of these patients. The dietary plan should follow a healthy diet model, in which the portion size is personalised according to the patient’s requirements and weight gain. Standardisation against personalised caloric prescriptions may confer advantages by facilitating accelerated early weight gain and lower the incidence of bed rest without increasing the incidence of RS [41].

No association between different nutrient contents (e.g. high-protein diet, diets with higher omega-3 polyunsaturated fatty-acid content, low-sodium versus normal-sodium diets) and refeeding outcome has been identified [18,42].

According to each protocol, oral nutritional supplements (ONS) is added if the patients’ nutritional requirements are not met, or their weight is stagnating. However, some groups use ONS since the first day of hospital admission in order to shorten the hospital stay. Complete hypercaloric polymeric diets, with a caloric density of 1.5 kcal/ml or even higher, are generally used [27,29,34,38,39,40].

Tube feeding (TF) is indicated in those patients with severe malnutrition who refuse to eat [36]. Some patients with AN may reject this treatment, especially in the beginning, considering it as an aggression that increases their sense of lack of control over their own diet. Usually, polymeric, lactose free and fibre-enriched formulas are employed. The administration rate is progressively increased to favour tolerance. The use of infusion pumps allows a better control and it impedes the manipulation of the treatment by the patient.

Percutaneous endoscopic gastrostomy (PEG) remains a limited choice of refeeding, however, it may be necessary in patients who need a prolonged treatment. In a study conducted by Born et al. there was no increase in the rate of complications reported for patients using PEG feedings in comparison with those receiving TF [43].

Parenteral nutrition (PN) is used even less in patients with AN, although, it could be a solution in patients with severe malnutrition in whom the digestive tract is not functional (digestive haemorrhage, intestinal obstruction, intestinal perforation, ileus, etc.) [44]. PN is associated with more severe complications than TF, and there is a need to tailor the formulas with special emphasis on volume, macronutrient distribution, micronutrients, and minerals supplementation. There have been published cases with patients treated with home PN [45].

In any of the refeeding modalities, a close supervision by the ward staff is required. A detailed protocol of the steps to follow improves the daily management of the treatment of these patients and may facilitate the weight gain process.

### 3.4. Physical Activity Programmes

The addition of physical activity in the treatment of patients with AN is exponentially growing, even though these programmes have been characterised as controversial [46,47,48]. Excessive exercise is often suggested as a causal factor in AN [47], and thus, it is not often prescribed in their clinical management [48]. However, prolonged restraint from physical activity may contribute to decreased bone mass, increased risk of atherosclerosis, and decreasing compliance with the treatment programme [49]. An increasing body of literature demonstrates that individuals suffering from AN could benefit from supervised exercise sessions in combination with nutritional assistance.

Several studies that integrated exercise programmes in the treatment of AN did not interfere with weight gain [50,51,52,53] or body fat [49] progression. Different types of exercises have been suggested as beneficial. For example, individualised yoga treatment resulted in reduced food preoccupation after each session [53]. A light resistance exercise programme for a period of 8 weeks showed positive effects on physical strength, body composition, and psychological well-being of hospitalised patients with AN [54]. Furthermore, a high-intensity resistance exercise training programme effectively and safely improved participants’ muscular strength as well as their ability to execute daily tasks [47]. In general, a programme incrementally going from mild to moderate exercise speed should be implemented, followed by the interrogation of emotions and thoughts after exercise [46].

Other benefits included less obligatory attitudes and distorted feelings towards exercise [51], less irregular or disorganised eating patterns [55], less drive for thinness, and reduction of the frequency of dangerous eating behaviours such as laxative abuse [56]. In addition, advantages include increased compliance with treatment [49], improved body satisfaction, positive mood states, and quality of life [52]. Physical strength [54] and cardiovascular fitness [48] also improve.

Notably, patients should only undertake physical training once weight stabilisation and nutritional status have progressed sufficiently in caloric and nutritional consumption in order to be able to support the chosen activities [46,54].

To date, there is no consensus or guideline for use of exercise in the treatment of AN [57]. The majority of studies were of small sample size and suggested training programmes varied, thus restricting generalisability of the findings. Moreover, the interventions are relatively short; there is limited follow-up, and most lack an assessment of the participants’ fitness.

Nonetheless, when performed in a therapeutic setting, where training is supervised, exercise is safe and may improve treatment outcomes in some AN patients.

### 3.5. Models of Organisation of the Nutritional Treatment

There is a need for a multidisciplinary team in the organisation model that may differ between centres. Regardless of this, having a written protocol may help to standardise treatment and improve clinical outcome. The protocol should establish the functions of each member of the team and it will determine the options each step of the way.

Close staff supervision is needed during hospitalisation, especially during mealtimes. Some authors have identified two main types of patterns: Rule adherence and rule bending when describing how staff choose how to intervene in different situations [58].

In the day hospital, the patients receive care without being hospitalised, including medical treatment, nutritional therapy, psychiatric and psychological care in individual and/or group modality, occupational therapy and social support. This resource of treatment may be indicated in some outpatients prior or after hospitalisation [59].

Continuity of care has proven to be useful to improve long term outcomes in these patients [60].

### 3.6. Nutritional Support Related Outcomes

The main clinical outcomes related to the refeeding process in patients with AN are weight gain, the length of hospitalisation and rehospitalisation rate, RS, and mortality. A summary of the nutritional support related outcomes published in different studies is shown in Table 6.

### 3.7. Weight Gain

The weight gain is the main objective in the nutritional rehabilitation of patients with AN. Achieving this is possible with any of the modalities of nutritional treatment. Some studies show that the greater the weight achieved by the patient up to hospital discharge, the lower the likelihood of relapse. This usually translates to a longer hospitalisation stay [6,63].

In a systematic review which included 10 studies with TF in AN patients, this treatment was considered safe and well tolerated, and effectively enhanced caloric intake and rate of weight gain in patients with AN [64].

In another systematic review, which included 7 observational studies with a total of 403 patients (children and adolescents), the prescribed calorie range varied between 1000–1900 kcal/day with progressive increase during hospitalisation. Additional TF increased the maximum energy intake and led to greater interim or discharge weight; however, this was also associated with a higher incidence of adverse effects [65].

Lastly, in the most recent systematic review by Hale et al., 19 out of 22 studies reported that significant short-term weight gain was achieved when TF was used for refeeding malnourished AN patients; however, results varied in the long-term weight gain, maintenance and recovery [66].

### 3.8. Hospital Stay and Rehospitalisation

Hospital stay as an outcome is closely related to the weight gain. In many cases, the length of hospitalisation is carefully monitored by the hospital management. One of the strategies to shorten the hospitalisation period in patients with AN includes the use of more aggressive refeeding consisting of diets with lower carbohydrate load (<40% total calories), in continuous administration, accompanied by an adequate supplementation of phosphate and potassium [37,67].

In a retrospective study including 2015 children and adolescents with AN, the authors refer a rehospitalisation rate of 3.8% and 17.2% after 30 days and 1 year after discharge, respectively [32]. 

In an RCT study with TF versus OD in AN, the results in terms of weight gain and rehospitalisation rate were better in patients with enteral nutrition [25].

In another RCT aimed to compare the effectiveness of hospitalisation for weight restoration to medical stabilisation in adolescent AN patients, the number of rehospitalisations and their respective length of stay (after the first hospital admission), within 12 months, were similar in the two groups when the treatment included family-based therapy [68].

### 3.9. Refeeding Syndrome

RS has been described in malnourished patients with AN and mainly involves mineral deficiencies (hypophosphataemia, hypokalaemia and hypomagnesaemia), as well as vitamin (thiamine) deficiencies, and volume overload. It can be presented as a symptomatic or asymptomatic clinical case, which implies that a close monitoring of laboratory parameters is necessary, especially during the first week of refeeding. The RS has been described with all feeding modalities (OD, TF, and PN) [66].

Table 7 summarises the main risk factors for developing a RS. There is no clear consensus on supplementing phosphorus in all patients starting refeeding, just in those who develop hypophosphataemia or in those severely malnourished [67].

Hypophosphataemia has been described as a hallmark in studies on RS. In a systematic review of 17 publications, an incidence of hypophosphataemia of 14% (0–38%) was reported, with the degree of malnutrition at admission being the main risk factor, without finding correlation with the refeeding rate (kcal/day) [34,69].

In a case-control study with 123 AN patients, the prevalence of hypophosphataemia was 33%, with the nadir occurring at the second day of admission. In this study, higher haemoglobin was the only risk factor associated with higher odds of developing hypophosphataemia; meanwhile a higher BMI, a higher serum potassium, and a higher serum prealbumin were protective factors against the development of hypophosphatemia [28].

### 3.10. Mortality

Classically, AN has been associated with a high mortality within the psychiatric disorders. In a series of 484 patients followed for 13 years, the authors refer a mortality rate of 1.2% [63].

In most of the revised articles in inpatients, the authors do not refer mortality rates during the refeeding process, even though the patients were severely malnourished [25,26,27,28,31,34,36,44].

On the other hand, in a retrospective study performed in France in 30 randomly selected ICUs including 68 patients with AN (average BMI at admission of 12 ± 3 kg/m^2^), the authors mention a RS rate of 10% and a mortality rate of 10%. The causes included acute respiratory distress syndrome and multiorgan-failure associated with major hydro-electrolytic problems [33].

As exemplified in the refeeding of other malnourished patients, a higher awareness of the risk associated with refeeding may decrease the risk of mortality [70,71].

## 4. Discussion

The treatment of AN patients is challenging, especially regarding weight gain recovery, which is the most important part of it. In severely malnourished patients, closely related to the weight gain is the risk of RS, which must be accordingly monitored. Moreover, there are factors that interfere with the recovery process, which are mainly related to the patient´s attitudes and fears.

There is a high variability in the treatment between centres, both in the feeding rate and type of nutritional treatment. One of the reasons is the lack of good scientific evidence in this topic. 

There are two established pathways when feeding the patients: “start low, advance slow” versus “start higher, advance faster”. Both ways have proven to be safe and effective in terms of the in appearance of RS and weight recovery, respectively [25,27,29,40,44]. However, the main difference between them is the length of hospital stay and costs. On this basis, some centres prefer the “start higher, advance faster” method in patients without severe malnutrition.

Regarding the refeeding process, all modalities of feeding (mainly OD, ONS, and TF) can be used in a safe and effective way. In a recent systematic review, no significant differences were found between TF and oral refeeding cohorts regarding gastrointestinal disturbance, RS, or electrolyte abnormalities. However, TF has proved a slight superiority achieving a higher weight at discharge and in the short term [66].

The reviewed literature does not show a high rate of RS during the treatment of these patients, probably due to a close supervision of the feeding process. However, hydro-electrolytic alterations especially hypophosphataemia is frequently reported and up to 40%. Moreover, these studies show that the degree of malnutrition at hospitalisation is the main risk factor for developing hypophosphatemia and it is not related to the refeeding rate [34,69]. Hypophosphatemia is mainly reported in the first days of refeeding. There is no agreement on the use of phosphate supplement as a prophylactic option or after an already established hypophosphatemia.

Patients with less than 70% of IBW, may benefit out of a slow refeeding process with additional mineral supplements because of their potential high risk of RS. However, in patients with higher body weight a faster refeeding rate could be an option in order to rapidly improve weight gain and shorten the hospital stay.

Even though mortality during hospitalisation is rarely reported, this fatal outcome cannot be neglected in the short term nor in the long-term [33].

As a novelty in the management of these patients, physical activity may play an important role. Supervised exercise seems to be safe and may improve treatment outcomes in some patients. 

This article contains a large number of studies and systematic reviews focused on the refeeding process of AN patients; however, the main limitation of the review is the quality of the studies. Most of them were observational, case-control, and only two were RCTs. This lowers the strength of recommendation of the results.

Evidence-based standards for clinical practice with clear outcomes (weight gain rate, hospital stay and rehospitalisation, RS, mortality) are needed to improve the management of these patients and standardise the healthcare process.

## 5. Conclusions

This review focuses on evidence of the refeeding process of malnourished inpatients with AN with emphasis on the clinical outcome (weight gain, hospital stay and rehospitalisation, RS and mortality). Firstly, patients with AN frequently present malnutrition and usually are at high risk of RS. Secondly, literature shows that RS risk mostly depends on the baseline nutritional status and is not associated with the refeeding rate and the type of nutritional treatment. Thirdly, TF seems to have better outcomes, especially in the short-term, though more studies are needed. Lastly, evidence-based standards for clinical practice with coherent outcomes are required to improve the management of these patients and standardise the healthcare process.

## Figures and Tables

**Table 1:** Diagnostic criteria of anorexia nervosa according to DSM-5 classification.

Anorexia nervosa	Restriction of energy intake relative to requirements leading to a significantly low body weight in the context of age, sex, developmental trajectory, and physical health. Significantly low weight is defined as a weight that is less than minimally normal or, for children and adolescents, less than that minimally expected. Intense fear of gaining weight or becoming fat, or persistent behaviour that interferes with weight gain, even though at a significantly low weight. Disturbance in the way in which one’s body weight or shape is experienced, undue influence of body weight or shape on self-evaluation, or persistent lack of recognition of the seriousness of the current low body weight.

**Table 2:** Clinical manifestations of anorexia nervosa.

Cardiovascular	Bradycardia and hypotension due to alterations in the autonomic nervous system. Electrocardiographic alterations: Atrial, ventricular arrhythmias and alterations in the QTc space. Myocardial alterations: decrease in cardiac mass, prolapse of the mitral valve and pericardial effusion.
Gastrointestinal	Delayed gastric emptying and constipation. Alterations of liver function tests. Parotid hypertrophy, loss of tooth enamel, gastroesophageal reflux, esophagitis, and oesophageal complications, Mallory–Weiss syndrome (especially in patients with purging habits, due to the chronicity of vomiting).
Neurological	Cortical atrophy and ventricular dilatation alterations. Alterations in psychological and cognitive tests.
Renal and Hydro electrolytic	Decrease in glomerular filtration rate. Hypokalaemia and hypochloremic metabolic alkalosis (vomit-induced) Metabolic acidosis (laxative abuse). Hyponatremia (diuretics abuse, potomania). Hypophosphatemia, hypokalaemia, and hypomagnesemia (refeeding syndrome, RS).
Bone	Osteopenia. Osteoporosis. Osteomalacia.
Endocrinological	Hypogonadotropic hypogonadism. Hypometabolism (resting energy expenditure, REE). Hypercortisolism, without the stigmas of Cushing’s syndrome. Euthyroid sick syndrome (low-normal range of T4 and TSH, with low levels of T3 and an increase in rT3). Hypoglycaemia.Growth and developmental delay in children.
Haematological	Bone marrow hypoplasia with gelatinous transformation presenting variable degrees of anaemia, leukopenia, and thrombocytopenia.
Dermatological	Russell’s sign. Xerosis. Lanugo. Hypercarotenaemia.

**Table 3:** Criteria for hospitalisation in anorexia nervosa.

**Medical Indications**	
Adults	Bradycardia < 40 bpm or tachycardia > 110 bpm. Blood pressure < 90/60 mmHg. Symptomatic hypoglycaemia. Hypokalaemia < 3 mmol/l. Hypothermia < 36.1 °C. Dehydration. Uncontrolled vomiting or purging behaviour. Weight < 75% of ideal body weight. Rapid loss of weight (several kgs in a week). Lack of improvement or worsening despite treatment as an outpatient.
Children and adolescents	Bradycardia < 50 bpm. Orthostatic hypotension (increase of >20 bpm in heart rate or drop in blood pressure > 10–20 mmHg with orthostatism). Blood pressure < 80/50 mmHg. Hypokalaemia or hypophosphataemia Rapid weight loss even if the weight is >75% of the ideal body weight. Symptomatic hypoglycaemia. Lack of improvement or worsening despite treatment as an outpatient.
**Psychiatric Indications**	
All ages	Suicidal ideation. Serious concomitant psychiatric illness. Impossibility to eat independently or needs tube feeding. Unfavourable family environment. Lack of cooperation despite treatment as an outpatient.

**Table 4:** Guidelines for the refeeding of malnourished anorexia nervosa patients.

Guideline	Population	Kcal/kg
United Kingdom: National Institute for Health and Clinical Excellence (NICE), 2017 [24]	Adults	5–20
United Kingdom: MARSIPAN: Management of Really Sick Patients with Anorexia Nervosa, 2014 [20]	Adults	5–20
United Kingdom: Junior MARSIPAN: Management of Really Sick Patients under 18 with Anorexia Nervosa, 2012 [21]	<18 years	15–20
American Psychiatric Association (APA), 2006 [6]	Adults	30–40
American Dietetic Association (ADA), 2006 [22]	Adults	30–40
Australia and New Zealand, 2004 [23]	Adults	15–20 (600–800 kcal/d)

**Table 5:** Example of a refeeding process in terms of macronutrient and micronutrient intake [19].

Days	Recommendations
Day 1–3	Start with 10–15 kcal/kg (600–1000 kcal/day). Prophylactic electrolyte supplementation (P, K, Mg). Thiamine (200–300 mg/day). Vitamins (200% RDI). Minerals and trace elements (100% RDI). Restrict the contribution of fluids to a zero balance. Restrict sodium to <1 mmol/kg/day. Glucose and electrolyte levels and the appearance of oedema should be adequately monitored, since the highest risk of RS occurs in these early days.
Day 4–10	Calorie intake will increase to allow weight gain, continuing with electrolyte, vitamin and mineral supplementation and close monitoring.

**Table 6:** Studies in patients with anorexia nervosa reporting clinical outcomes of the nutritional treatment.

Study	Study Type	Nº Patients	Mean Age (years)	Weight (kg) or BMI (kg/m^2^) at Admission	Weight (kg) or BMI (kg/m^2^) at Discharge	Length of Stay (days)	Kcal/kg or kcal/day at Admission	Type of Nutritional Treatment	Outcome
Rigaud et al., 2007 [25]	RCT a: TF b: control	a: 41 b: 40	a: 22.5 b: 24.2	a: 12.1 b: 12.8	a: 17.9 b: 15.9	60	a: 1000 (D0)–2450 (D14) b: 1000 (D0)–1850 (D14)	a: OD + TF b: OD	No RS 1-year relapse: a: 44% b: 52%
Diamanti et al., 2008 [44]	Retrospective	a: 104 b: 94	15	a: 36.3 b: 41	a: 39.6 b: 41.5	a: 30.7 b: 15.6	40 kcal/kg	a: OD + PN b: OD	No RS a: Hypophosphataemia (6 cases), Hypopotassaemia (3 cases)
Gentile et al., 2010 [26]	Retrospective	33	22.8	11.3	13.5	60	1408	OD + TF + iv. glucose	No RS
Vignaud et al., 2010 [33]	Retrospective	68	31	12	-	7.6 (in ICU)	22.3 kcal/kg	OD + TF + TPN	RS (10%) Mortality (10%)
Whitelaw et al., 2010 [34]	Retrospective	29	15.7	72.9% IBW	-	-	1900–2200 (89% of patients)	OD + ONS + TF (7 patients)	Mild hypophosphataemia (37%)
Garber et al., 2012 [35]	Prospective	35	16.2	16.3	-	17	1205	OD	No RS Hypophosphataemia (20%)
Gentile et al., 2012 [36]	Retrospective	10	22	11.2	17.3	90	1199	OD + TF + iv. glucose	No RS
Agostino et al., 2013 [37]	Retrospective cohort study: a: Nasogastric cohort b: bolus-fed cohort	165	14.9	a: 16.6 b: 16.7	-	a: 33.8 b: 50.9	a: 1617 b: 1069	a: 31 patients b: 134 patients	No RS a: Hypopotassaemia (1 case); readmissions at 6 months (12.9%) b: Hypopotassaemia (1 case); mild-moderate hypophosphataemia (8 cases); readmissions at 6 months (23%)
Garber et al., 2013 [38]	Prospective cohort: a: high calorie b: low calorie	56	16.2	a: 16.6 b: 15.8	-	14.9	a: 1700 b: 1093	OD + ONS	No RS Hypophosphataemia (45%) Length of stay: a: 11.9 days b: 17.6 days
Golden et al., 2013 [39]	Retrospective a: low calorie b: high calorie	a: 88 b: 222	16.1	a: 15.9 b: 16.1	a: 17.2 b: 17.1	a: 16.6 b: 13	a: 1163 b: 1557	OD + ONS + TF (occasionally)	No RS Hypophosphataemia (15.8%) Hypomagnesaemia (15.2%) Hypopotassaemia (20%)
Leclerc et al., 2013 [40]	Retrospective	29	14.7	16.4	-	35.8	1500	OD + ONS	Hypophosphataemia (3.5%)
Hofer et al., 2014 [27]	Retrospective	65	27.9	13.7	15	49.5	10 kcal/kg	OD (mostly) OD + ONS (8.1%) OD + TF (8.1%) TF (5%) OD + TPN (1.2%)	Mild RS (10.5%) Severe hypopotassaemia (4.7%)
Brown et al., 2015 [28]	Retrospective case-control	123	28	13	13.9	13	1200	OD	Hypophosphataemia (33%)
O’Connor et al., 2016 [61]	RCT a: intervention b: control	a: 18 b: 18	13.8	a: 32.4 b: 34.6	a: 34.1 b: 35.6	10	a: 38 kcal/kg/d b: 16 kcal/kg/d	OD + TF	a: Hypophosphataemia (28%) b: Hypophosphataemia (11%)
Marugán et al., 2016 [29]	Retrospective	50	14.5	15.45	17.58	44.54	1000	OD ONS (16%) TF (8%)	No symptoms of RS
Kameoka et al., 2016 [30]	Retrospective	99	30.9	<17.5	-	82.7	Low calorie diet	OD	Hypophosphataemia (21.2%)
Smith et al., 2016 [62]	Retrospective	129	15.8	15.8	17.1	14.9	1585	OD + ONS	No RS Hypophosphataemia (47.3%) Hypokalaemia (12%)
Davies et al., 2017 [31]	Retrospective	65	24	12.8	14.4	60	20-30	OD, ONS (infrequently)	Mild hypophosphataemia (<0.8 mmol/L) (6.5%) Hypokalaemia (1 case). No RS
Peebles et al., 2017 [32]	Retrospective	215	15.3	17.1	18.2	11	1466	OD, TF (10%)	No RF. Hypophosphataemia (14%) 3.8% and 17.2% rehospitalisations after 30 days and 1 year, respectively

TF: Tube Feeding, OR: Oral Diet, ONS: Oral Nutritional Supplements, RS: Refeeding Syndrome, PN: Parenteral Nutrition, TPN: Total Parenteral Nutrition, RCT: Randomised Controlled Trial.

**Table 7:** Identification of patients with high risk of refeeding syndrome.

Patients with 1 or more of the following:	BMI < 16 kg/m^2^ Unintentional weight loss of >15% in the previous 3–6 months. Minimum or no intake for >10 days. Low levels of K, P, or Mg before refeeding.
Or, patients with 2 or more of the following:	BMI < 18.5 kg/m^2^ Unintentional weight loss of >10% in the previous 3–6 months Minimum or no intake for >5 days. History of alcohol abuse, drugs, insulin treatment, chemotherapy, antacids, or diuretics.
